# Surface Profiling and Core Evaluation of Aluminum Honeycomb Sandwich Aircraft Panels Using Multi-Frequency Eddy Current Testing

**DOI:** 10.3390/s17092114

**Published:** 2017-09-14

**Authors:** Tyler Reyno, P. Ross Underhill, Thomas W. Krause, Catharine Marsden, Diane Wowk

**Affiliations:** 1 Department of Mechanical and Aerospace Engineering, Royal Military College of Canada, Kingston, ON K7K 7B4, Canada; diane.wowk@rmc.ca; 2 Department of Physics, Royal Military College of Canada, Kingston, ON K7K 7B4, Canada; ross.underhill@rmc.ca (P.R.U.); thomas.krause@rmc.ca (T.W.K.); 3 Faculty of Engineering and Computer Science, Concordia University, Montreal, QC H3G 1M8, Canada; c.marsden@concordia.ca

**Keywords:** eddy current, multi-frequency eddy current, surface profiling, surface damage, honeycomb paneling

## Abstract

Surface damage on honeycomb aircraft panels is often measured manually, and is therefore subject to variation based on inspection personnel. Eddy current testing (ECT) is sensitive to variations in probe-to-specimen spacing, or lift-off, and is thus promising for high-resolution profiling of surface damage on aluminum panels. Lower frequency testing also allows inspection through the face sheet, an advantage over optical 3D scanning methods. This paper presents results from the ECT inspection of surface damage on an approximately flat aluminum honeycomb aircraft panel, and compares the measurements to those taken using optical 3D scanning technology. An ECT C-Scan of the dented panel surface was obtained by attaching the probe to a robotic scanning apparatus. Data was taken simultaneously at four frequencies of 25, 100, 400 and 1600 kHz. A reference surface was then defined that approximated the original, undamaged panel surface, which also compensated for the effects of specimen tilt and thermal drift within the ECT instrument. Data was converted to lift-off using height calibration curves developed for each probe frequency. A damage region of 22,550 mm^2^ area with dents ranging in depth from 0.13–1.01 mm was analyzed. The method was accurate at 1600 kHz to within 0.05 mm (2σ) when compared with 231 measurements taken via optical 3D scanning. Testing at 25 kHz revealed a 3.2 mm cell size within the honeycomb core, which was confirmed via destructive evaluation. As a result, ECT demonstrates potential for implementation as a method for rapid in-field aircraft panel surface damage assessment.

## 1. Introduction

Impact damage in all-aluminum honeycomb sandwich structures, such as wing surfaces and internal floorboards, may produce permanent denting of the aluminum skin accompanied by a buckled honeycomb core that often remains bonded to the skin [1]. Both the dent and core damage produced by an impact affect the residual strength of a panel and, therefore, may compromise the structural integrity of the component, potentially leading to its failure [2]. Characterization of dent depth and the area of dent in aircraft support structures is a key element used in the determination of remaining panel lifetime. Methods of evaluating damage in aluminum honeycomb sandwich structures have included X-ray and optical shearography [3]. Portable methods for the assessment of honeycomb panel denting damage have involved dial or digital depth gauge [4,5,6,7,8,9,10,11], a ruler, or, in some cases, a high-resolution electronic indicator [12,13]. However, a disadvantage of these methods is that measurements are subject to interpretation and variation based on inspection personnel. Manual measurements may also require repetition to confirm observations. Creaform Inc. is developing and marketing a portable optical 3D scanning system for the assessment of hail damage on in-service aircraft. However, this technique does not offer the added benefit of inspecting core geometries in these panels. For the panel considered in this study, dents corresponding with barely visible impact damage (BVID) were of interest, which are typically the result of low-velocity impact.

Eddy current testing (ECT) is commonly used for the detection of surface breaking flaws in conductive materials [14]. Applications of ECT for flaw detection include piping and heat exchanger inspection of nuclear power plants [15,16,17]. For these applications, it offers advantages such as high sensitivity, rapid scanning, and flexibility [14]. ECT is also used to evaluate defects in various other environments and configurations [14]. These include flaws in steam generator tubes [18,19,20,21,22,23], fatigue cracks [24,25,26], corrosion [27,28,29,30,31,32,33], material microstructure near welds [34], and the estimation of pearlite percentages in plain carbon steel [35,36] and in cast iron [37]. High-density eddy current C-Scan data has also been used for the analysis of bolt hole eddy current [38] and estimation of micro-crack lengths [39]. For sub-millimetre applications, an array probe has been used for the imaging of surface-breaking defects in the bore holes of metallic parts [40]. The effectiveness and reliability of ECT for the detection of surface-breaking cracks in cast stainless steel reactor components has also been studied [41].

ECT has been applied to measure probe-to-specimen spacing, known as lift-off. Applications like paint and metal deposition thickness measurement are well known [42]. However, the potential for surface profiling is not as well recognized. In one case, eddy current probe output was combined with high-density C-Scan data to extract information regarding local deformations and wear on the inner surfaces of pressure tubes [43]. This application produced topographic images of a specimen; however, the study did not calibrate lift-off response and, therefore, did not quantify the size of features in the pipe. The extraction of surface damage measurements via ECT represents an attractive alternative to traditional depth gauge methods, since it can substantially reduce inspection time, store data for further analysis and retrieval, and makes use of technology and trained personnel that are already part of the aircraft maintenance program.

**Figure 1:**
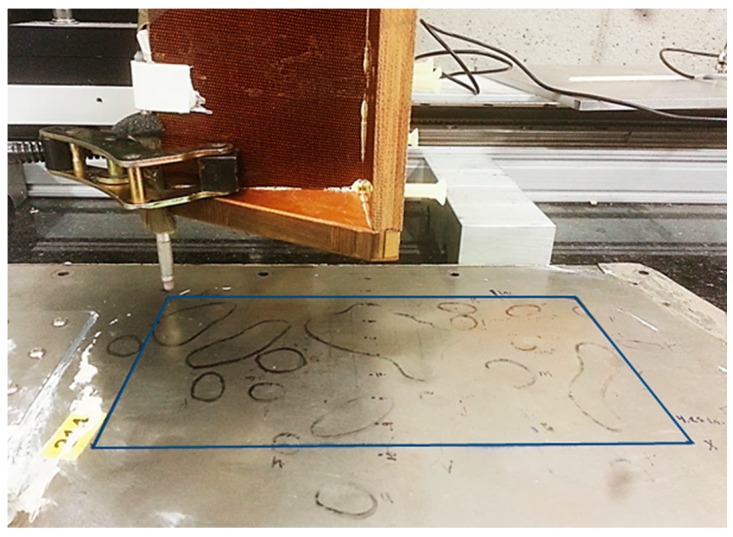
Experimental setup with outlined visual inspection results of sample.

This study investigated the feasibility of using eddy current testing, combined with high-density C-Scan information, as a method of evaluating surface damage due to denting on aluminum honeycomb sandwich aircraft panels. An ECT pencil probe was retrofitted to a servo controlled C-Scan apparatus and probe output was calibrated to lift-off (mm). Measurements were compared to those taken on the same panel using an optical 3D scanning method [44], as well as with identifications made by inspection personnel as shown on the panel in Figure 1.

## 2. Materials and Methods

Surface damage was quantified on a retired honeycomb sandwich panel that had been used in a military helicopter. This panel had properties as listed in Table 1.

A Nortec pencil probe, Z-145-P, which was attached via lever clamp to the C-Scan apparatus, was used to perform the ECT measurements. Prior to scanning, the panel was aligned with the scanning apparatus using alignment blocks placed at both ends of the panel to ensure equidistance from the scanning apparatus. Scanning was performed over a 205 mm × 110 mm damage region (Figure 1), which contained approximately 14 dent impressions, with depths ranging from 0.13–1.01 mm as measured via optical 3D scanning. Optical 3D scanning was performed using a FARO ScanArm^®^ measurement system mounted to a lab workstation with single-point repeatability of 0.024–0.064 mm. Subsequent analysis of the 3D scan data was performed using Geomagic Design X software (3D Systems, Rock Hill, SC, USA).

Eddy current signals were generated and received via an Olympus NDT MultiScan MS5800 eddy current instrument (Olympus, Quebec, QC, Canada). This instrument was used to perform input and output signal quadrature, displayed as Lissajous plots on an impedance plane as part of the data processing method, as well as handle the complex (real and imaginary) voltage input and output for the system. The operating frequencies for the probe were 25, 100, 400 and 1600 kHz.

TecView software (TecScan, Boucherville, QC, Canada) was used to control the automated scanning apparatus. This included defining the (X, Y) coordinate axes of the damage region for the C-scan, as well as specifying the path of the probe during the scanning process. Figure 1 shows the setup of the C-Scan apparatus, with reference to the probe and aircraft panel. The sampling interval of the C-Scan was set to 1 mm, which yielded 22,550 separate measurements. The raw output data was converted to lift-off (Z-axis) using height calibration curves developed prior to scanning.

The ECT acquisition software used in conjunction with the MS5800 eddy current instrument was MultiView 6.1 (Olympus, Quebec, QC, Canada). Probe setting specification and control were performed within MultiView; namely, starting and stopping probe input, and presenting output data from the probe in real-time (strip chart display). Balancing of the probe was likewise conducted in MultiView with the probe located at an undented point on the panel surface. Nulling at this location meant that the change in signal observed when moving over a dent impression could be interpreted as lift-off. In addition, the output signal (or locus), the response to lift-off, was aligned in MultiView display software such that the lift-off component of the signals was aligned with the Y-axis of the C-Scan output.

The operating frequencies and voltage of the probe were selected such that signal saturation was not observed and eddy currents would not penetrate deeper than the 0.5 mm thickness of the upper Al skin layer at the highest frequency of 1600 kHz. For the given specimen, through-thickness penetration would skew and complicate the surface profiling results based on the complex interior of the honeycomb sandwich panel, which included adhesive and crushed aluminum honeycomb core. However, testing at lower frequencies, including 25 kHz, offered the added potential of inspecting the core geometry of the panel. Using relative permeability µ_r_ = 1 and electrical conductivity σ = 32% IACS = 1.89 × 10^7^ S/m for Al 7075-T6 [45], theoretical depths of penetration were calculated at the four testing frequencies using Equation (1) from [45] as tabulated in Table 2.

Depth of Penetration, δ = 1/√(πfμσ),(1)

**Figure 2:**
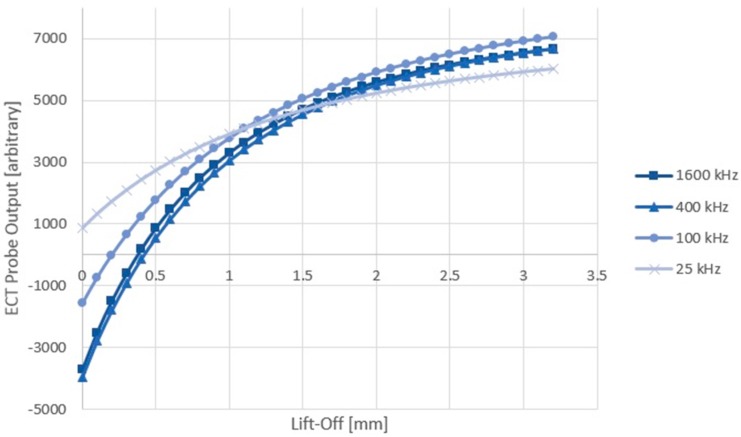
Eddy current testing (ECT) calibration curves at various frequencies; probe output vs. lift-off (mm).

A calibration test was performed prior to the C-Scan process, which produced curves for converting the probe output data to lift-off (mm) at the four frequencies, simultaneously. Note that only the 1600 kHz signal gave a penetration, 3δ that was less than the face sheet thickness (3δ = 0.27 mm, which was smaller than the 0.5 mm face sheet thickness). The lower frequency results with 3δ being larger than the thickness of the face sheet may be invalid due to sensing of face sheet thickness variations or honeycomb structure beneath the face sheet. This test was performed in the same location as the probe balance point within the damage region. Data was extracted at a 0.1 mm resolution over a lift-off range of 3.2 mm (Z-axis) starting from contact. This produced 33 data points, as shown in Figure 2, which were linearly interpolated to produce the calibration curves.

It was necessary to define a new reference surface for the eddy current lift-off measurements at each frequency, which approximated the original, undamaged surface of the panel. This is because the 2D C-Scan plane did not exactly match the plane of the aircraft panel, which demonstrated some curvature. The new reference surface for each frequency was thus three-dimensional in nature, defined using Equation (2), where Z(x,y) and Z′(x,y) represent the original and adjusted eddy current measurements, respectively. The values of A, B, C and D were chosen to minimize the residual sum square (RSS) difference between the reference surface and the adjusted eddy current measurements at the edges of the damage region.

Z′(x,y) = (Z(x,y) − (AX + BY + CXY + D),(2)

**Figure 3:**
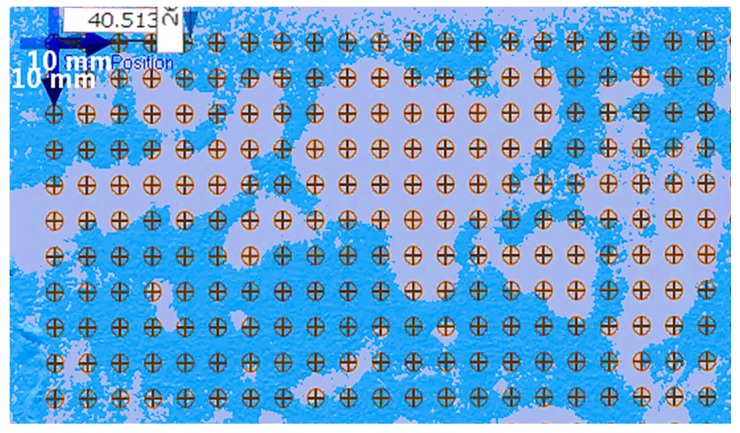
11 × 21 grid of comparison points for damage region as evaluated within 3D scanning software; blue patterned background is 3D scan point cloud data.

To validate the eddy current results, an 11 × 21 grid of measurement points with 10-mm spacing was exported from the optical 3D scanning software to compare with the corresponding eddy current measurements made within the damage region. This grid of comparison points was specified as shown in Figure 3 within the 3D scanning software.

## 3. Results

The process of defining a reference surface for the eddy current measurements yielded coefficient values for Equation (2) as shown in Table 3 for each frequency.

**Figure 4:**
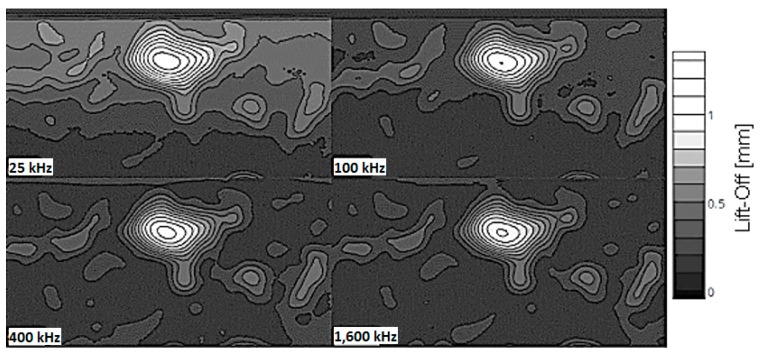
Four frequency comparison.

Figure 4 shows a visual comparison of the surface contour plots developed for the eddy current measurements at each frequency.

**Figure 5:**
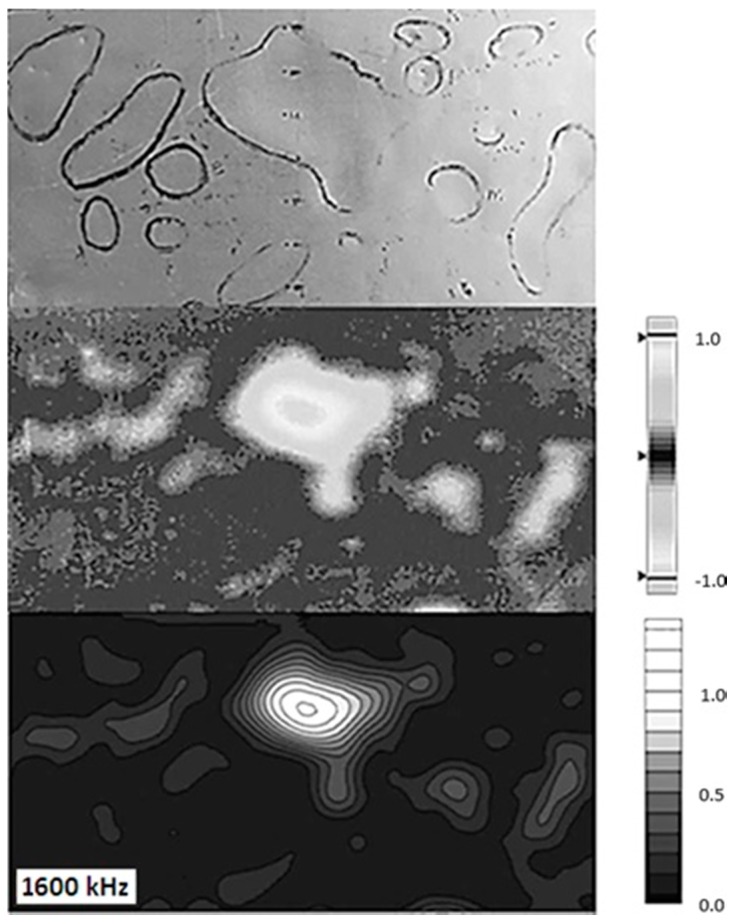
Damage region (**top**); 3D scanning deviation map (**middle**); 1600 kHz ECT contour map (**bottom**).

Figure 5 shows a comparison between a photograph of the damaged panel surface with demarcated dent regions, the 3D scanning results, and a contour map of the 1600 kHz eddy current measurements. Note the comparatively higher resolution obtained from the 1 mm × 1 mm sampling grid of the ECT C-Scan.

**Figure 6:**
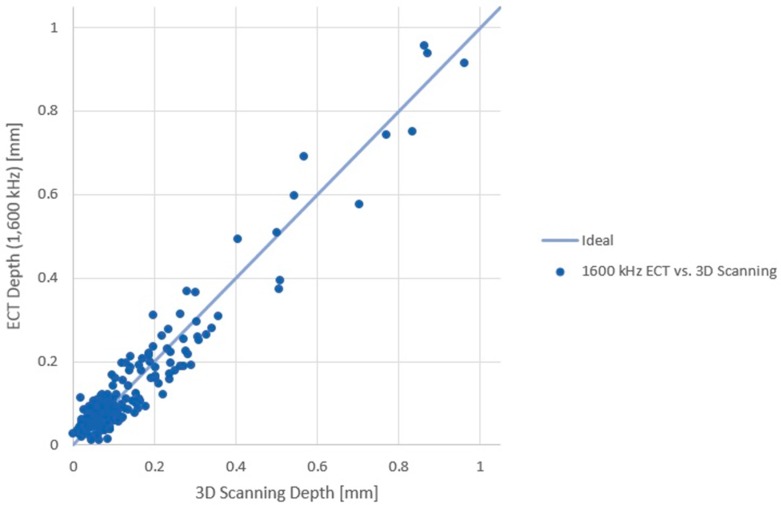
Dent depth (ECT) vs. dent depth (3D scanning method).

Error was evaluated to two standard deviations between the eddy current results, at each frequency, and optical 3D scanning measurements at 231 points, as tabulated in Table 4. Maximum absolute difference reported the maximum difference calculated between the ECT and 3D scanning results. A depth (ECT) versus depth (3D scanning) plot was developed, as shown in Figure 6, for the 1600 kHz data, where the line represents the ideal case of the ECT results being equal to the 3D scanning results.

## 4. Discussion

Of the four frequencies, the 1600 kHz results were the most accurate in terms of surface profiling capability, matching the maximum dent depth (1.01 mm) identified within the damage region, while also yielding the lowest error to two standard deviations (0.05 mm) and maximum absolute difference (0.14 mm) compared with the 231 optical 3D scanning measurements.

**Figure 7:**
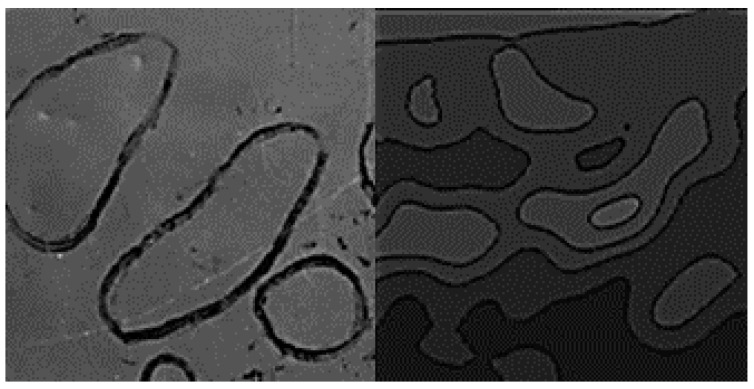
Dent identification comparison; inspection personnel (**left**) vs. ECT method (**right**).

The ECT results also demonstrated potential for improved capability for dent characterization compared with visual methods. This was seen within the portion of the damage region shown in Figure 7, where the contour map of the eddy current measurement results varied from the identifications made by visual inspection personnel. This is valuable for accurately measuring dent lengths and areas, which are target parameters of aircraft panel surface inspections. These parameters may also be evaluated using the described ECT method by performing image processing of the contour maps obtained from the ECT measurement results.

**Figure 8:**
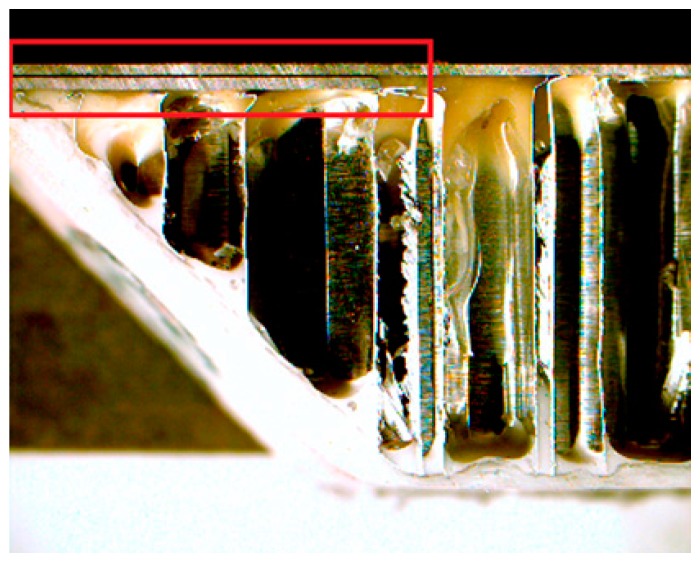
Second layer of aluminum identified under top face sheet near top of damage region close to panel flange.

Compared with other surface damage inspection methods like optical 3D scanning, multi-frequency eddy current testing offers the additional benefit of internal inspection capability for aluminum honeycomb sandwich panels. This includes identification of variable thickness regions and evaluation of honeycomb core geometries. For instance, the eddy current measurements compiled at 25 and 100 kHz revealed a second layer of aluminum beneath the top face sheet at the top of the damage region. This was confirmed by cutting the panel, which revealed the second layer as shown in Figure 8.

**Figure 9:**
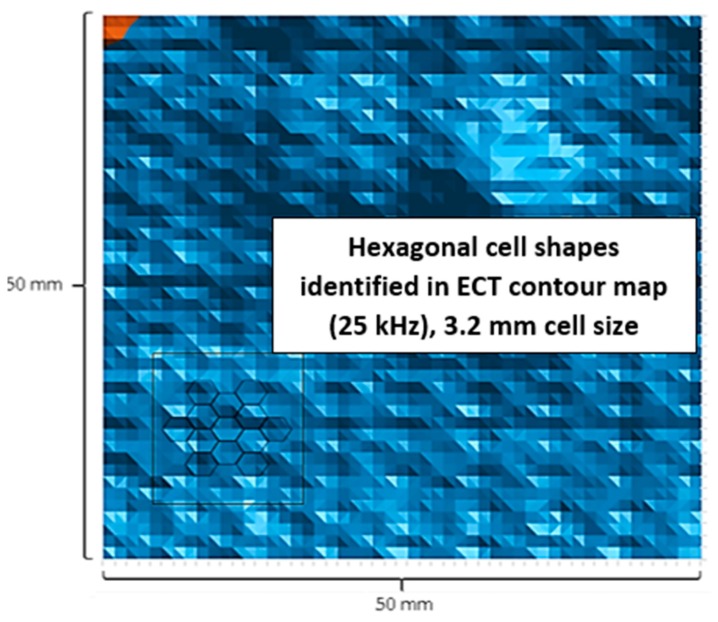
ECT core evaluation with identified cell geometries (25 kHz).

**Figure 10:**
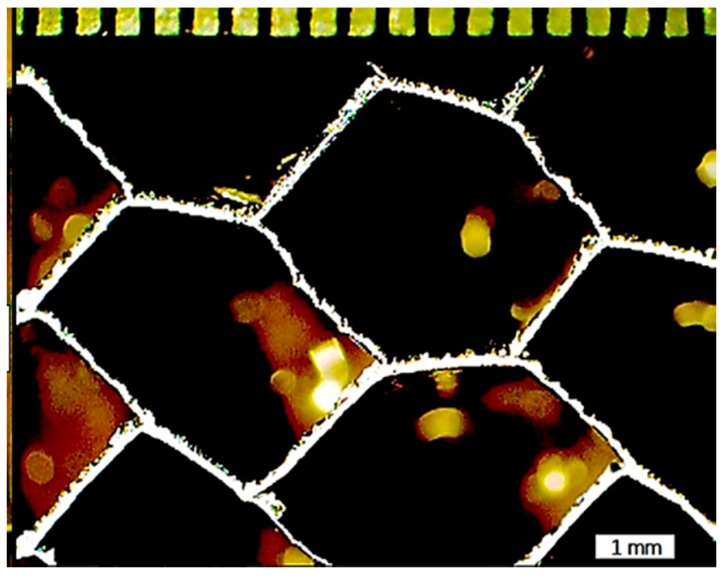
Honeycomb core cell geometry obtained from destructive examination. Demarcations at top of figure are half mm intervals.

**Figure 11:**
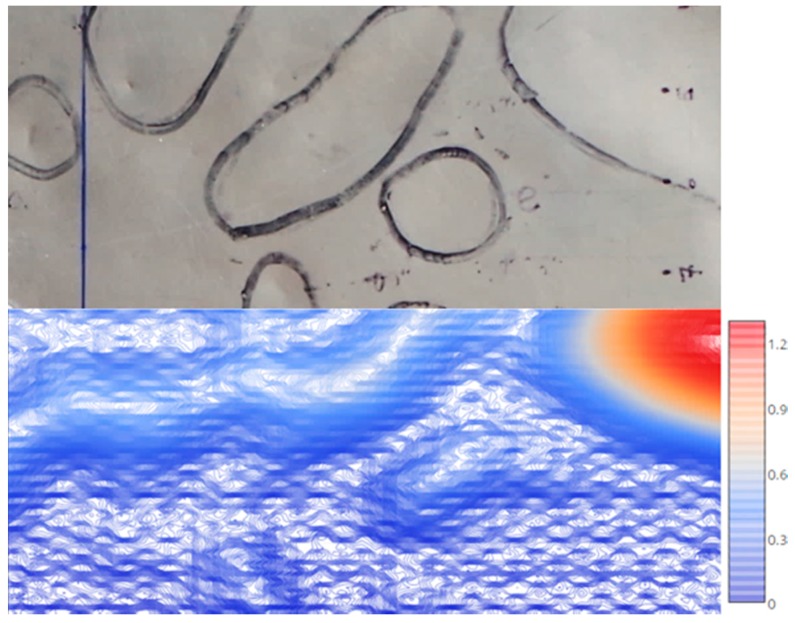
ECT core evaluation; panel surface (**top**) and ECT results plotted at 0.0015 mm contour step size (**bottom**) (25 kHz).

Plotting the low 25 kHz frequency results likewise allowed characterization of the hexagonal cell geometries located beneath the top face sheet, shown in Figure 9. Measurement of the contours confirmed the honeycomb core cell size as 3.2 mm, which was performed using image processing. Here, the hexagonal cell shapes were identified based on contouring, and the scale of the image was defined by the known perimeter dimensions of the damage region. The measured 3.2-mm cell size agreed with destructive examination as shown in Figure 10. Linear contour plotting of the 25 kHz results also enabled investigation of the core as shown in Figure 11 at a step size of 0.0015 mm compared to the same region observed visually.

There is potential for in-field implementation of this method using portable eddy current technologies. These would likely involve scanning of aircraft surfaces with a handheld eddy current array system combined with encoder information. For the evaluation of entire panel surfaces, it is assumed that the described ECT method would decrease inspection time due to automated extraction and compilation of damage geometry information, and would enhance the reliability of the measurements. Greater measurement density may also be achieved using ECT compared to the 3D scanning method, where it was observed that the output capability of the ECT method (22,550 measurements) was two magnitudes higher than that achievable using the 3D scanning software, but this may be a limitation of the particular 3D scanning software that was implemented.

## 5. Conclusions

ECT was investigated as a method of surface profiling for an all-aluminum honeycomb sandwich aircraft panel. Comparison of the method at 1600 kHz with 231 optical 3D scanning measurements demonstrated that the eddy current results were accurate to within 0.052 mm (two standard deviations). Lower frequency testing at 25 kHz also proved feasible for the evaluation of internal core geometries. ECT-based methods hold the potential for rapid and reliable means of measuring dent depths, lengths and areas. As a result, this novel technique has the potential to provide timely extraction of surface damage measurements for on-site component examination.

## Figures and Tables

**Table 1:** Panel specifications.

Property	Specification
Top face sheet material	Al 7075-T6
Core material	Al 5052
Bottom face sheet material	Epoxy/fiberglass
Adhesive	Heat-resistant epoxy Hysol^®^ EA 934NA
Total panel thickness	12.7 mm (0.50″)
Top face sheet thickness	0.51 mm (0.020″)
Core thickness	11.7 mm (0.46″)
Cell size	3.2 mm (0.125″)
Cell wall thickness	0.025 mm (0.001″)

**Table 2:** Depth of penetration analysis.

Frequency (kHz)	Theoretical Depth of Penetration, δ (mm)	% Penetration (3δ) into Exposed Face Sheet Thickness
25	0.7	400
100	0.4	200
400	0.2	100
1600	0.1	50

**Table 3:** Equation (2) coefficient values. Residual sum square (RSS).

Frequency (kHz)	Coefficient, Equation (2)				Resultant RSS Difference
	A	B	C	D	
25	−0.18	0.38	0.0011	−0.19	0.0029
100	−0.23	0.069	0.0014	0.16	0.0065
400	−0.24	−0.13	0.0015	0.30	0.056
1600	−0.22	−0.10	0.0013	0.28	0.042

**Table 4:** Error analysis.

Frequency (kHz)	Error (Two Standard Deviations) (mm)	Maximum Absolute Difference (mm)
25	0.240	0.40
100	0.069	0.16
400	0.056	0.15
1600	0.052	0.14
